# Pathogenesis of Olfactory Disorders in COVID-19

**DOI:** 10.3390/brainsci12040449

**Published:** 2022-03-27

**Authors:** Laura Ziuzia-Januszewska, Marcin Januszewski

**Affiliations:** 1 Department of Otolaryngology, Central Clinical Hospital, Ministry of Interior and Administration, 02-507 Warsaw, Poland; 2 Department of Obstetrics and Gynecology, Central Clinical Hospital, Ministry of Interior and Administration, 02-507 Warsaw, Poland; lek.med.mjanuszewski@gmail.com

**Keywords:** olfactory disorders, loss of smell, anosmia, COVID-19, SARS-CoV-2, pathogenesis

## Abstract

Since the outbreak of the SARS-CoV-2 pandemic, olfactory disorders have been reported as a frequent symptom of COVID-19; however, its pathogenesis is still debated. The aim of this review is to summarize the current understanding of the pathogenesis of smell impairment in the course of COVID-19 and to highlight potential avenues for future research on this issue. Several theories have been proposed to explain the pathogenesis of COVID-19-related anosmia, including nasal obstruction and rhinorrhea, oedema of the olfactory cleft mucosa, olfactory epithelial damage either within the olfactory receptor cells or the supporting non-neural cells (either direct or immune-mediated), damage to the olfactory bulb, and impairment of the central olfactory pathways. Although the pathogenesis of COVID-19-related anosmia is still not fully elucidated, it appears to be mainly due to sensorineural damage, with infection of the olfactory epithelium support cells via the ACE1 receptor and disruption of the OE caused by immense inflammatory reaction, and possibly with direct olfactory sensory neurons infection mediated by the NRP-1 receptor. Involvement of the higher olfactory pathways and a conductive component of olfactory disorders, as well as genetic factors, may also be considered.

## 1. Introduction

Since the outbreak of the SARS-CoV-2 pandemic, smell impairment has been reported as a frequent symptom of COVID-19, with reported prevalence ranging widely in the literature from 5 to 98.3% [1,2,3,4,5,6,7,8,9,10,11]. The association of olfactory disorders (OD) with COVID-19 is now established, but OD pathogenesis is still debated [12,13,14,15].

Odor detection begins with the binding of odorant molecules to odor receptors localized on the dendritic cilia of the olfactory sensory neurons (OSNs) in the olfactory epithelium (OE) [16,17]. OSNs axons cross the skull base through the cribriform plate and form synapses within the olfactory bulb (OB) [16,17]. Olfactory information is sent from the OB to higher brain centers [16,17].

The OE is a complex tissue consisting of multiple cell types, including OSNs, sustentacular (SUS) cells, mucus-secreting Bowman’s gland cells, microvillar cells, and stem cells including globose and horizontal basal cells. In addition, macrophages and dendritic cells are present in the OE [18,19,20]. SUS cells act to structurally support OSNs, protect OSNs by phagocyting and/or detoxifying potentially harmful agents, enable odor detection by endocytosis of the olfactory binding protein and odorant complex, supply OSNs with glucose necessary for high energy olfactory transduction cascade, and maintain local fluid and electrolyte balance [17,18,21]. The basal cells can differentiate to replace OSNs during normal turnover or injury [19,20]. Bowman’s glands secrete mucus, containing water, salts, mucin glycoproteins, enzymes, antibodies, and odorant binding proteins (OBPs), which transport the hydrophobic odorant molecules through the mucus to the OSNs cilia [20]. Olfactory dysfunctions can be classified into three types: conductive disorders caused by obstruction of the nasal cavities and subsequent blockage of odorant transmission to the olfactory epithelium (OE), sensorineural loss caused by damage of the OE or olfactory nerve, and central dysfunction resulting from damage to the olfactory processing pathway in the central nervous system (CNS) [16,22].

Loss of the sense of smell due to upper respiratory tract infections (URTI) is primarily considered a conductive loss secondary to rhinorrhea and mucosal edema, and usually normalizes as the infection resolves [23]. However, in some cases, loss of smell may persist after the resolution of UTRI, suggesting a sensorineural disorder known as post-viral olfactory dysfunction (PVOD) [23,24]. PVOD is one of the leading causes of anosmia in adults, accounting for approximately 11–40% of cases [24,25,26]. According to previous studies, several respiratory viruses can cause PVOD, including rhinovirus, parainfluenza virus, Epstein–Barr virus and some coronaviruses, with previously discovered coronaviruses accounting for 10–15% of cases [24,25,27].

Several theories have been proposed to explain the pathogenesis of COVID-19-related anosmia. These include conductive loss of smell due to nasal obstruction and rhinorrhea, oedema of the olfactory cleft mucosa that prevents odorants from reaching the olfactory epithelium, olfactory epithelial damage, infection of the olfactory nerves and, through retrograde neuroinvasion, the olfactory bulb, and impairment of the central olfactory pathways. due to direct viral invasion or indirect injury caused by hypoxia, endothelial damage, or an abnormal inflammatory response. Damage to the olfactory epithelium may be caused by direct viral invasion of the olfactory sensory neurons (OSN), most likely mediated by the neuropilin-1 receptor, or by infection of the non-neuronal cells of the olfactory epithelium, leading to horizontal viral spread to OSNs or impaired morphological and physiological support of OSNs [13,17,22,23,28,29,30,31].

## 2. SARS-CoV-2 Cellular Entry Mechanism

**Figure 1:**
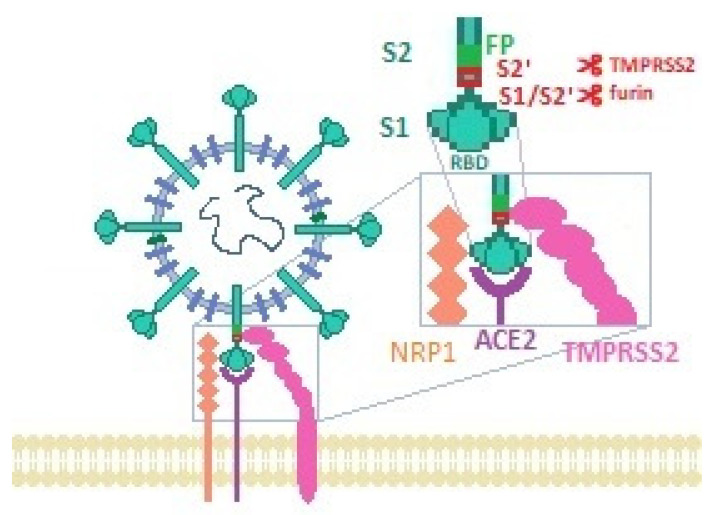
SARS-CoV-2 entry. Spike protein domain S1 is pre-activated by the host furin. The receptor binding domain (RBD) of S1 binds to the ACE2 receptor. Cleavage at S2′ site by the type 2 transmembrane protease (TMPRSS2) causes further structural changes in the spike protein and expose the fusion peptide (FP) of S2 that enables the fusion of the viral and host cells. The furin-cleaved S1 fragment of the spike protein may also bind directly to neuropilin-1 (NRP-1).

To discuss the pathogenesis of COVID-19-related anosmia, the mechanism of SARS-CoV-2 cellular entry must be elucidated. SARS-CoV-2, similarly to SARS-CoV, utilizes the S1 domain of its spike (S) protein for attaching the virion to the host cell membrane by binding to the host angiotensin-converting enzyme 2 (ACE-2) receptor [32,33]. This interaction requires cleavage and priming of the S protein by cell proteases, including transmembrane protease serine 2 (TMPRSS2) and furin, which allows fusion between the cellular and the viral membranes, and viral entry into the cell via endocytosis [17,32,34,35]. Alternative SARS-CoV-2 entry mediators have also been suggested, including receptors and cofactors such as CD147 and neuropilin-1 (NRP1), and activators such as cathepsin Figure 1 [36,37,38]. ACE-2 is expressed in multiple cell surfaces throughout the body, including lung parenchyma, respiratory epithelium, gastrointestinal epithelium, endothelium, arterial smooth muscles, neuronal glial cells, neurons, renal tubular cells, heart, and lymphoid tissues [33,39,40,41,42]. TMPRSS2 was also found to be expressed in multiple organs, such as in the respiratory tract, salivary glands, gastrointestinal tract, liver, and kidneys [34,43]. This ubiquitous expression of ACE2 and TMPRSS2 may explain the pleiotropic effects of SARS-CoV-2 infection [44]. However, the primary targets of SARS-CoV-2 are the cells manifesting high expression of these proteins, especially those that co-express ACE2 and TMPRSS2, such as respiratory and olfactory epithelial cells [17,38]. It is noteworthy that SARS-CoV-2 appears to have a particularly strong affinity for ACE2, estimated to be 10-to-20-fold higher than for SARS-CoV, which might explain its particular impact on chemosensory systems [29,33,45].

## 3. The Conductive Pathomechanism of COVID-19-Related Anosmia

Many studies have reported the early onset [2,5,46] and early recovery [4,5,7,8,47,48] of olfactory disorders (OD) in the course of COVID-19. This could argue in favor of a conductive mechanism for OD, related to the inflammation of nasal mucosa [9,47]. However, many COVID-19 patients report OD in the absence of nasal obstruction and rhinorrhea [3,5,6,10,48]. In a study by Chung et al. [47], only 17% of patients with OD reported nasal symptoms, and minimal inflammatory infiltrates were found in nasal biopsy specimens. Moreover, many studies have reported no association between the presence and severity of OD in COVID-19 and nasal symptoms [6,49], and in a study by Lechien et al. [49], some anosmic COVID-19 patients had normal acoustic rhinometry. In addition, Haehner at el. [50], found that self-rated changes in nasal airflow were more pronounced in SARS-CoV-2 negative patients with smell loss compared to SARS-CoV-2 positive patients with OD, while smell deterioration was reported to be more severe by SARS-CoV-2 positive individuals. These findings suggest that rhinitis and nasal mucosa obstruction are not the main factors in the development of COVID-19-related OD.

## 4. Olfactory Cleft Edema

Some imaging studies in anosmic COVID-19 patients show olfactory cleft mucosal edema that may prevent odorants from reaching the olfactory epithelium, even in the absence of nasal congestion [51,52,53]. However, other reports did not reveal significant olfactory cleft obstruction [54,55,56], suggesting that it is not the primary mechanism in COVID-19-related anosmia [12,55,56].

## 5. Infection of Higher Olfactory Pathways

Another possible pathomechanism for anosmia is the neural hypothesis, suggesting direct damage to the olfactory nerves, and a retrograde invasion of the olfactory bulb and higher olfactory pathways in the CNS [57,58].

### 5.1. The Neuroinvasive Potential of SARS-CoV-2

The neuroinvasive potential of SARS-CoV-2 is supported by various neurological manifestations of COVID-19 described in the literature [1,59,60,61]. In a retrospective case series by Mao et al. [1], 36.4% of 214 SARS-CoV-2-positive patients had neurologic manifestations, including dizziness, headache, impaired consciousness, acute cerebrovascular disease, ataxia, seizure, smell or taste impairment, vision impairment, nerve pain, and symptoms of skeletal muscle injury manifestations [1]. In another retrospective study of 814 COVID-19 patients, 57.4% developed neurological symptoms, including myalgias, headache, dizziness, disorders of consciousness, myopathy, dysautonomia, cerebrovascular diseases, seizures, movement disorders, encephalitis, Guillain-Barré syndrome, and optic neuritis [61]. Furthermore, encephalopathy, meningitis, acute transverse myelitis, and coma have also been reported in the literature [59,60]. It has also been hypothesized that viral infection of the respiratory center in the medulla oblongata could cause respiratory failure, which may even occur in the absence of dyspnea [33,41]. Moreover, a study by Bhattacharjeea et al. [62], found that COVID-19 patients have significantly reduced olfactory matching abilities, which the authors considered a sign of cognitive impairment, possibly related to infection in the higher brain centers.

However, the neurological symptoms of COVID-19 do not necessarily indicate a direct viral neuroinvasion, as they may also be due to hypoxic brain injury, cerebrovascular injury, or immune mediated damage [59,63,64]. Indeed, severe SARS-CoV-2 pneumonia may lead to systemic hypoxia and hypercapnia, peripheral vasodilatation, and anaerobic metabolism with an accumulation of toxins and subsequent brain damage due to cerebral edema [60,63]. Cerebrovascular injury may result from viral binding to endothelial ACE2 receptors, which in turn leads to increased luminal pressure and intracranial hemorrhage. Moreover, cerebrovascular injury may be due to an abnormal inflammatory response known as the cytokine storm, which involves the overproduction and excessive release of cytokines, including IL-1β, IL-6, CXCL10, and TNFα, and increased activation of T lymphocytes, macrophages, and endothelial cells, leading to a vascular leakage and an overactivation of the complement system and the coagulation cascade, with subsequent disseminated intravascular coagulation, thromboembolism, and multiorgan failure [59,63,64]. Interestingly, IL-6, which has been shown to regulate neuronal and glial cell activity, may play a role in regulating olfactory neuronal activity, and can also directly inhibit olfactory function through activating apoptosis using TNF-α, or affecting signaling by neuropoietin [65]. In a study by Cazzolla et al. [65], OD was correlated with higher levels of IL-6, and improvement of the olfactory function was associated with decreased IL-6 levels [65]. However, Sanli et al. [66], reported significantly lower serum IL-6 levels in COVID-19 patients with OD compared to normosmic subjects, so the role of IL-6 in the pathogenesis of OD remains unclear.

Some studies reported the presence of SARS-CoV-2 particles in post-mortem brain examination [67,68] and SARS-CoV-2 RNA was detected by RT-PCR in cerebrospinal fluid samples [60], supporting the direct brain injury hypothesis, although the brain seems to contain either the least volume of viral particles of all of the sampled tissues, or no particles at all [29,68], and RT-PCR positivity does not necessarily prove the presence of whole viral particles in the cerebrospinal fluid (CSF) [29].

Direct invasion of the CNS could occur either through the hematogenous or the retrograde neuronal route [1]. In the hematogenous pathway, viruses may reach the bloodstream due to increased permeability of local blood vessels and epithelial disruption, and may then enter the brain by one of several ways: invasion of endothelial cells of the blood-brain-barrier (BBB), paracellular transmigration enabled by increased BBB permeability caused by the release of inflammatory mediators, crossing of the blood–CSF barrier in the choroid plexus, or infection of leukocytes capable of passing through the BBB (the “Trojan horse” mechanism) [54,69,70,71]. However, according to Li et al. [41], the hematogenous route of SARS-CoV spread is unlikely since almost no viral particles were detected in the non-neuronal cells of infected brain areas [41,54], which may also be true for SARS-CoV-2 [72,73].

### 5.2. The Transneuronal Route of CNS Involvement

The olfactory pathway was hypothesized as a potential route for direct viral neuroinvasion. In this mechanism, the virus reaches the brain through the olfactory epithelium by invading peripheral nerve terminals and propagating via axonal transport towards the OB, from where it may spread trans-synaptically using retrograde and anterograde transport to other brain areas [1,59,63,74,75]. Alternatively, the virus may pass from the OE through the olfactory ensheathing cells directly to the CSF surrounding the olfactory nerve bundles and the OB [20,45].

This transneuronal route of CNS involvement is supported by the post-mortem examinations revealing SARS-CoV-2 viral particles and related damage to be more present in the OSNs and the OB than in the brainstem [76,77]. Interestingly, it has also been hypothesized that inflammatory infiltration of the OB with increased INF-I levels may be responsible for the development of anosmia, and at the same time contributes to the arrest of viral spread into the brain [75]. The hypothesis of neuronal damage as the causative mechanism in SARS-CoV-2-related OD is further supported by reports of OB abnormalities in magnetic resonance imaging (MRI), including features of micro bleeding and oedema, observed in anosmic COVID-19 patients [78,79]. Moreover, transient cortical hyperintensity in the right gyrus rectus has been described [78]. Other studies have also shown the reduction in OB volumes, indicative of its atrophy [53,80]. On the other hand, Akkaya et al. [81] found no significant difference in OB volumes between anosmic and normosmic COVID-19 patients. However, they observed an association between OB morphology and OD, with normal, oval or inverted J-shaped OBs (type N) more common in normosmic patients, while shrunken or flattened OBs (type R) and the presence of asymmetric contour lobulation or more than one hyperintense focus on T2 images (type D) were dominant in the anosmia group [81]. In another study, Esposito et al. [82] found no significant difference in OB volume between the previously SARS-CoV-2-infected hyposmic patients and healthy controls; however, diffusion and functional MRI revealed an increase in neural connectivity within the olfactory cortex and functional connectivity of the anterior piriform cortex, indicating a characteristic brain connectivity response in COVID-19-related hyposmia.

Moreover, several animal studies on previous human coronaviruses support the neuronal route of infection [74,83]. HCoV-OC43 was reported to be found in mouse OSNs and OB 3 days after intranasal inoculation and in the brain 4 days post infection [84]. Moreover, in a study using K18-hACE2 transgenic mice expressing human ACE2, Netland et al. [81] demonstrated that intranasal infection with SARS-CoV resulted in neuroinvasion through the OB and rapid viral spread to the brain regions connected to the OB, such as the piriform and infralimbic cortices, the basal ganglia, and the midbrain, with significant neuronal death [54,83]. Similarly, intranasal administration of SARS-CoV-2 in mice with the human ACE2 gene caused a rapid infection of the brain [85]. Additionally, Jiao et al. [86] demonstrated that in rhesus monkeys, SARS-CoV-2 RNA was detectable in the CSF, the olfactory trigone, and the entorhinal area on days 1, 4, and 7 after intranasal inoculation, respectively.

However, it should be noted that the studies in mouse models utilized transgenic mice with human ACE2 that could be ectopically expressed in many cells, including OSNs, and therefore may not be a reliable model of the viral tropism [28,29]. Indeed, several sequencing studies found ACE2 and TMPRSS2 to be co-expressed in non-neuronal cells of the OE rather than in the OSNs [29,38,87]. Moreover, sequencing and immunostaining studies revealed that OB neurons also do not express detectable levels of ACE2 [17,29]. Similarly, no significant expression of ACE2 or TMPRSS2 was found in neurons in the brain [17,29].

Moreover, the higher incidence of anosmia compared with CNS symptoms, as well as the commonly observed early recovery of OD, argue against the central mechanism of SARS-CoV2-related OD [47,58,88,89].

## 6. Damage to the Olfactory Neuroepithelium

Another mechanism for COVID-19-related anosmia may be sensorineural damage, with the disruption of the OE.

Indeed, Vaira et al. [88], reported massive olfactory epithelium disruption in a patient with anosmia persisting for three months after SARS-CoV-2 infection. Similarly, de Melo et al. [13], in a study of the olfactory neuroepithelium of seven COVID-19 patients presenting with acute loss of smell, reported the SARS-CoV2 infection in multiple cell types within the OE, including OSNs, support cells, and immune cells. Interestingly, sampling of the olfactory mucosa of the patients with long-term anosmia revealed the persistence of virus transcripts up to 6 months after initial diagnosis, accompanied by the protracted inflammation [13]. Moreover, the presence of SARS-CoV-2 in OSNs was shown in intranasally infected Syrian hamsters [90].

As mentioned above, several sequencing studies found ACE2 and TMPRSS2 to be co-expressed in non-neuronal cells of the olfactory epithelium, including sustentacular (support) cells, stem cells, and perivascular cells, rather than in the OSNs [17,38,87]. It was therefore hypothesized that SARS-CoV-2 infects high-ACE2-expressing non-neuronal cells of the OE before passing to OSNs [45]. Infection of OSNs may result from horizontal viral spread from the adjacent support cells or from dissemination of the virus within the OE after its tissue architecture is disrupted by inflammatory infiltrates [13]. Of note, in the aforementioned study by Netland et al. [83], SARS-CoV was detected in the OB after approximately 60 h post inoculation, and subsequent transport in the brain only took a further 12–20 h, suggesting that initial replication and accumulation took place within the OE before the neural invasion [6,45,83]. In addition, in a study by de Melo et al. [13], infection of the neuroepithelium was associated with the loss of OSN cilia, which are necessary for odor detection and transduction [13,28], and recovery of olfactory function was observed after the restoration of cilia in the late phase of infection [13]. Furthermore, Zazhytska et al. [91], in a study of SARS-CoV-2-infected hamsters and humans, found no depletion in OSNs, but observed significant and persistent, non-cell autonomous downregulation of the olfactory receptor and their signaling pathway genes, preceded by reorganization in OSNs nuclear architecture, indicating that the virus may alter the physiology of OSNs without their direct infection.

Moreover, as SUS cells play an important role in the structural and functional support of the OSNs, it appears that their infection could cause impaired olfactory function even without direct neuronal invasion, by architectural OE damage and lack of physical support, impaired signaling, ionic imbalance, and initiating an immune response [17,22,28,29,30]. Indeed, a study by Bryche et al. [28] in a golden Syrian hamster model demonstrated that, after intranasal instillation, SARS-CoV-2 infected SUS cells, with immense infiltration of the immune cells, massive and rapid desquamation of the OE and a significant loss of OSN cilia, but the virus was not detected in the OSNs or OB [28]. This hypothesis is also in agreement with the relatively rapid recovery of most patients, occurring within weeks after infection, which may reflect the regenerative capability of the SUS cells. The infection of OE stem cells, including horizontal basal cells (HBCs), may be responsible for longer lasting OD in some cases [29,30]. A noteworthy study by Torabi et al. [92] of mucosal samples taken from COVID-19 patients found increased levels of proinflammatory cytokine TNF-α, and the activation of an antiviral signaling cascade in the OE is hypothesized in the literature as reducing the expression of odorant receptors in OSNs [89]. Moreover, the involvement of Bowman’s gland can deteriorate mucus production, further affecting odor detection [29]. Indeed, as olfactory receptors turn over every 24 h, but do not contain blood and lymphatic vessels or exhibit mitosis, their generation and maturation depend on stem cells activated by growth or transcription factors secreted into nasal mucus from nasal serous glands. SARS-CoV-2 infects these serous glands, and therefore may cause inhibition of stem cell activity and subsequent loss of smell [14].

However, it should be noted that although almost no expression of ACE2 is found in the OSNs, they do express NRP1, which could account for direct OSNs infection [12,13,36]. Cantuti-Castelvetri et al. [36] found abundant expression of NRP1 in almost all cell types of the OE, and study of human autopsies revealed that SARS-CoV-2 infected NRP1-positive cells in the OE and OB. In addition, the same study reported NRP1-mediated transport of virus-sized particles into the CNS after intranasal inoculation in mice [36]. Moreover, the expression of NRP1 has also been found in neuronal progenitor cells [12,36], which could also play a role in the persistent anosmia observed in some COVID-19 patients [12].

Furthermore, although the previously reported rare occurrence of parosmia during recovery was considered to suggest the absence of damage to peripheral sensory neurons [89,93], it should be noted that its prevalence may have been overlooked in early reports, due to the short observation period [94]. In a study by Hopkins et al. [94], the prevalence of parosmia after a median interval of 2.5 months (range 0–6) from the onset of OD was as high as 43.1% [94], which may indicate the presence of disturbed regrowth and the domination of immature neurons in the OE [15]. Moreover, Di Stadio et al. [95] found that 40% of COVID-19 patients with persistent OD reported parosmia, and 16.6% of the parosmic individuals misperceived odors used during olfactory training. The authors hypothesized that SARS-CoV-2-induced inflammation of the neuroepithelium and OB may result in impaired olfactory recovery with aberrant OSNs regeneration and misperception of odors in the neuroepithelium, as well as altered olfactory receptor mapping in the OB. Therefore, stimulation of these inflamed areas during olfactory training could increase both olfactory recovery and parosmia [95].

## 7. Genetic Link to the Pathomechanism of OD

There may be also a genetic link to the pathomechanism of OD in COVID-19. Indeed, in a multi-ancestry genome-wide association study of COVID-19-related self-reported loss of smell or taste, Shelton et al. [96] identified a single associated locus in the vicinity of the UGT2A1 and UGT2A2 genes, encoding uridine diphosphate glucuronosyltransferase (UGT) enzymes. Interestingly, these enzymes are expressed in the OE and in rats UGT2A1 is involved in metabolizing odorants and olfactory signal termination [96,97,98]. This argues for a possible role of the identified genes in the dysfunction of OE cells and the associated olfactory disorders [96].

## 8. Conclusions

The pathogenesis of COVID-19 related anosmia is still not fully elucidated; however, it appears to be mainly due to sensorineural damage, with infection of the OE support cells via the ACE1 receptor and disruption of the OE caused by immense inflammatory reaction, and possibly with direct OSNs infection mediated by the NRP-1 receptor. Involvement of the higher olfactory pathways and a conductive component of OD, as well as genetic factors, may also be considered.

## Data Availability

Not applicable.
